# Traumatic myositis ossificans: multifocal lesions suggesting malignancy on FDG-PET/CT—a case report

**DOI:** 10.1007/s00256-020-03521-w

**Published:** 2020-06-25

**Authors:** Miho Sasaki, Yuka Hotokezaka, Reiko Ideguchi, Masataka Uetani, Shuichi Fujita

**Affiliations:** 1 Department of Radiology and Cancer Biology, Nagasaki University Graduate School of Biomedical Sciences, 1-7-1 Sakamoto, Nagasaki, 852-8588 Japan; 2 Department of Radioisotope Medicine, Nagasaki University Graduate School of Biomedical Sciences, 1-7-1 Sakamoto, Nagasaki, 852-8588 Japan; 3 Department of Radiological Sciences, Nagasaki University Graduate School of Biomedical Sciences, 1-7-1 Sakamoto, Nagasaki, 852-8588 Japan; 4 Department of Oral Pathology, Nagasaki University Graduate School of Biomedical Sciences, 1-7-1 Sakamoto, Nagasaki, 852-8588 Japan

**Keywords:** Myositis ossificans, Heterotopic ossification, Magnetic resonance imaging, Positron-emission tomography

## Abstract

Myositis ossificans (MO) is a benign soft-tissue lesion characterized by the heterotopic formation of the bone in skeletal muscles, usually due to trauma. MO is occasionally difficult to diagnose because of its clinical and radiological similarities with malignancy. We report a case of traumatic MO (TMO) in the masseter and brachial muscles of a 37-year-old man who presented with painless swelling in the left cheek and severe trismus. Due to the absence of a traumatic history at the first consultation and identification of a tumorous lesion in the left masseter muscle by magnetic resonance imaging (MRI), the lesion was suspected to be a malignant tumor. Subsequently, 18F-fluorodeoxyglucose positron-emission tomography/computed tomography (FDG-PET/CT) showed multiple regions of high FDG uptake across the whole body, suggestive of multiple metastases or other systemic diseases. However, intramuscular calcifications were also observed in the left masseter and brachial muscles, overlapping the areas with high FDG uptake. Moreover, multiple fractures were seen in the rib and lumbar spine, also overlapping the areas with high FDG uptake. Based on these imaging findings, along with a history of jet-ski trauma, TMO was suspected. The left cheek mass was surgically excised and histologically diagnosed as TMO. In this case report, FDG-PET/CT could detect multiple TMOs across the whole body. To the best of our knowledge, cases of multiple TMOs located far apart in different muscles are rare, and this may be the first report.

## Introduction

Myositis ossificans (MO) is a self-limiting, benign ossifying lesion, which classically presents with a “do not touch” lesion [1]. It is characterized by heterotopic ossification within muscles and soft tissues, and is most commonly observed in the large muscles of the extremities as solitary lesions [2, 3]. The term “traumatic MO” (TMO) is preferentially used when a traumatic episode is apparent [3].

TMO is the most common type of MO, accounting for approximately 60–75% of cases [4, 5]. When a patient with TMO reports a characteristic history of trauma and shows a clear calcification on imaging, the diagnosis is relatively uncomplicated. However, several recorded cases of MO have occurred in patients with no apparent history of trauma, and consequently, malignancy was suspected. The imaging features of MO are non-diagnostic, as they mimic other benign or malignant lesions, including bizarre parosteal osteochondromatous proliferation, melorheostosis, recurrent giant cell tumor, parosteal osteosarcoma, extraskeletal osteosarcoma, and soft tissue sarcoma [2, 3].

To the best of our knowledge, this is the first case of multiple TMOs found in distant muscle sites. Here, TMOs were detected in the masseter and brachial muscles using 18F-fluorodeoxyglucose positron-emission tomography/computed tomography (FDG-PET/CT). Although the multiple FDG uptake sites visible across the body were non-diagnostic and complicated the definitive diagnosis, FDG-PET/CT could detect multiple foci of TMOs in accordance with a history of trauma, CT imaging, and histopathological findings.

This case report addresses the appearance of TMO on FDG-PET/CT scans and demonstrates that FDG-PET/CT can detect multiple foci of trauma. Furthermore, a comprehensive medical interview, especially to document recent trauma, is fundamental to diagnose TMO as well as to avoid complicated and expensive tests.

## Case report

**Fig. 1:**
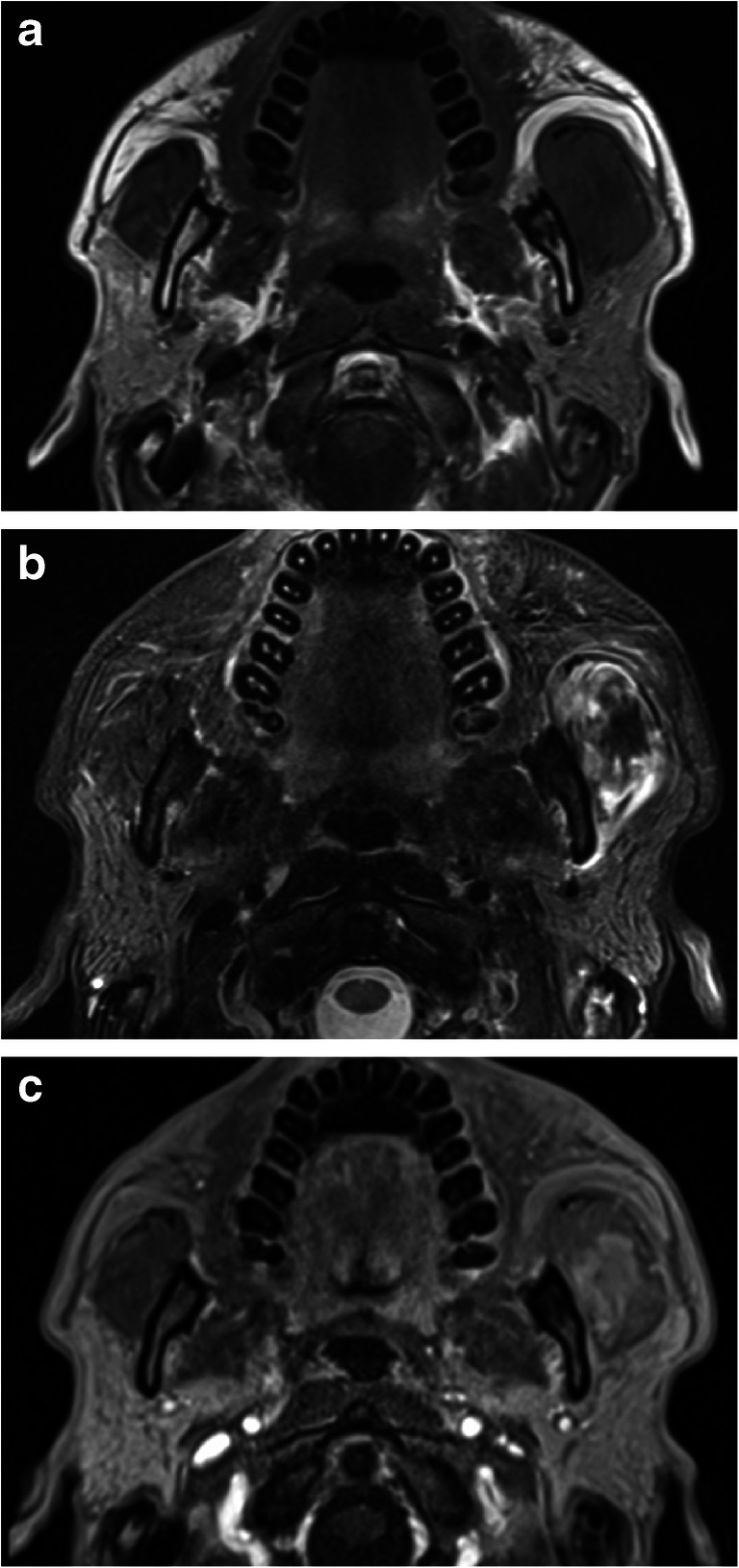
Axial magnetic resonance images. **a** The T1-weighted image shows swelling of the left masseter with isointensity to the muscle. **b** The fat-suppressed T2-weighted image shows swelling of the left masseter muscle with an apparent increase in signal intensity in the periphery and a focal mass-like or reticular low signal intensity in the center. **c** Dynamic contrast-enhanced image 30 s after injection of the contrast agent shows high enhancement in the central mass-like area of the left masseter muscle

A 37-year-old man was referred for further examination and treatment of swelling in his left cheek and severe trismus. He had noticed painless swelling and trismus over 2 weeks, with rapid progression over the preceding 2–3 days. Physical examination revealed a hard swelling of the left cheek with no pain or signs of acute inflammation. Additionally, a solid mass was palpable in the left masseter region. Panoramic radiography showed multiple root stumps; however, no apparent abnormal findings were observed in the left side of the mandible. Intraoral examination also revealed multiple root stumps without any associated symptoms. Blood tests revealed abnormally high levels of creatine phosphokinase (1593 U/L), while other markers including inflammatory signs were within a normal range. There was no relevant past medical or family history. As the previous doctor had suspected that the patient had a malignant tumor, magnetic resonance imaging (MRI) and FDG-PET/CT were performed. T1-weighted MRI revealed swelling of the left masseter isointensity to the muscle (Fig. 1a). On the fat-saturated T2-weighted image, the left masseter showed a reticular or mass-like low and peripheral high signal intensities (Fig. 1b). No abnormal or inflammatory signal intensities were apparent in the masticatory space around the left masseter muscle. A dynamic study after gadolinium contrast-enhancement revealed an increased enhancement in the central mass-like area (Fig. 1c), although time-intensity curves showed the same rapid uptake, followed by a gradual increment pattern in the various regions of interest placed in the left masseter muscle. The apparent diffusion coefficients from diffusion-weighted imaging were relatively low in the central mass-like region and intermediate-to-high in the periphery. These MRI findings were suggestive of an inflammatory process; however, combined with the clinical findings, the possibility of a malignant tumor could not be discarded.

**Fig. 2:**
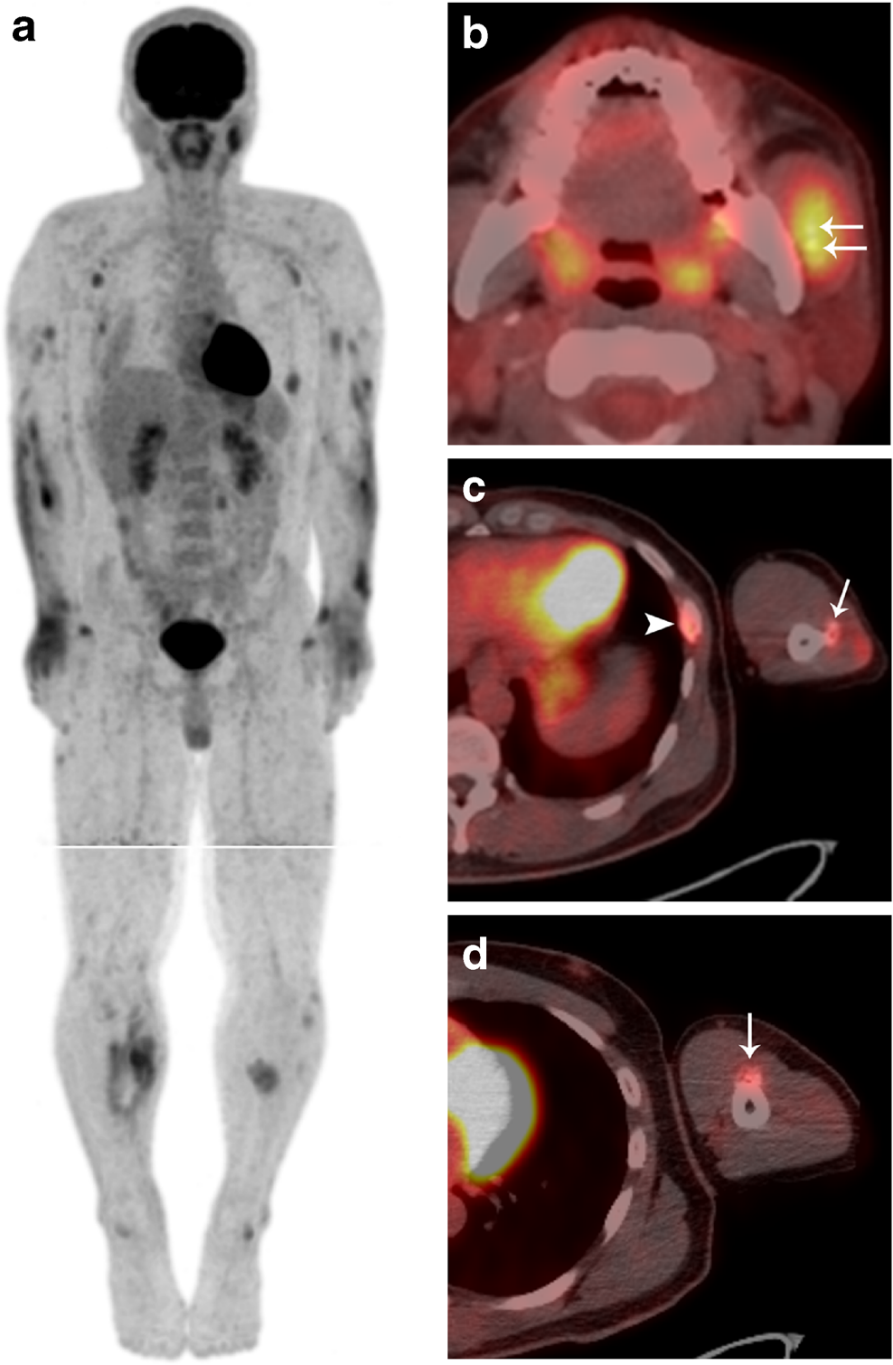
18F-fluorodeoxyglucose (FDG) positron-emission tomography/computed tomography (PET/CT) images. **a** The maximum intensity projection image shows multiple spotty or diffuse high-uptake regions across the whole body. The maximum standardized uptake values ranged from 2 to 8. In the lower limb, the image was acquired separately at a delayed scan. **b**, **c**, **d** PET/CT fusion images show small calcifications overlapping the FDG uptakes in the left masseter (**b**, arrows), triceps brachii (**c**, arrow), and biceps brachii (**d**, arrow) muscles. The maximum standardized uptake value of the left masseter muscle is 5.6 (**b**), and those of the left brachial muscles are 2.7 (**c**) and 2.3 (**d**), respectively. A fracture is seen in the left 8th rib, overlapping the FDG uptake (**c**, arrowhead)

On subsequent FDG-PET/CT, the left masseter muscle showed an increased FDG uptake (Fig. 2a, b). Furthermore, multiple spotty or diffuse high-uptake regions were observed throughout the whole body, including the upper and lower extremities, the left 8th rib, the left transverse process of L3, and the axillary lymph nodes (Fig. 2a). Most of the high-uptake regions in the upper and lower extremities had spread to the muscles and cutaneous adipose tissues. Hot spots in the left 8th rib (Fig. 2c) and the left transverse process of L3 corresponded with fractures. Small calcified foci were seen overlapping with the high-uptake regions of the left masseter (Fig. 2b), triceps brachii (Fig. 2c), and biceps brachii (Fig. 2d) muscles. The maximum standardized uptake value was approximately 6 in the left masseter muscle and ranged between 2 and 8 in other accumulated regions. Since some of the multiple hotspots corresponded with fractures, external multiple trauma was suspected; however, some types of malignancy, including lymphoma or multiple metastases, and systemic diseases, such as collagen disease or sarcoidosis, were also possible, although neither was fully explained by the FDG-PET/CT findings.

**Fig. 3:**
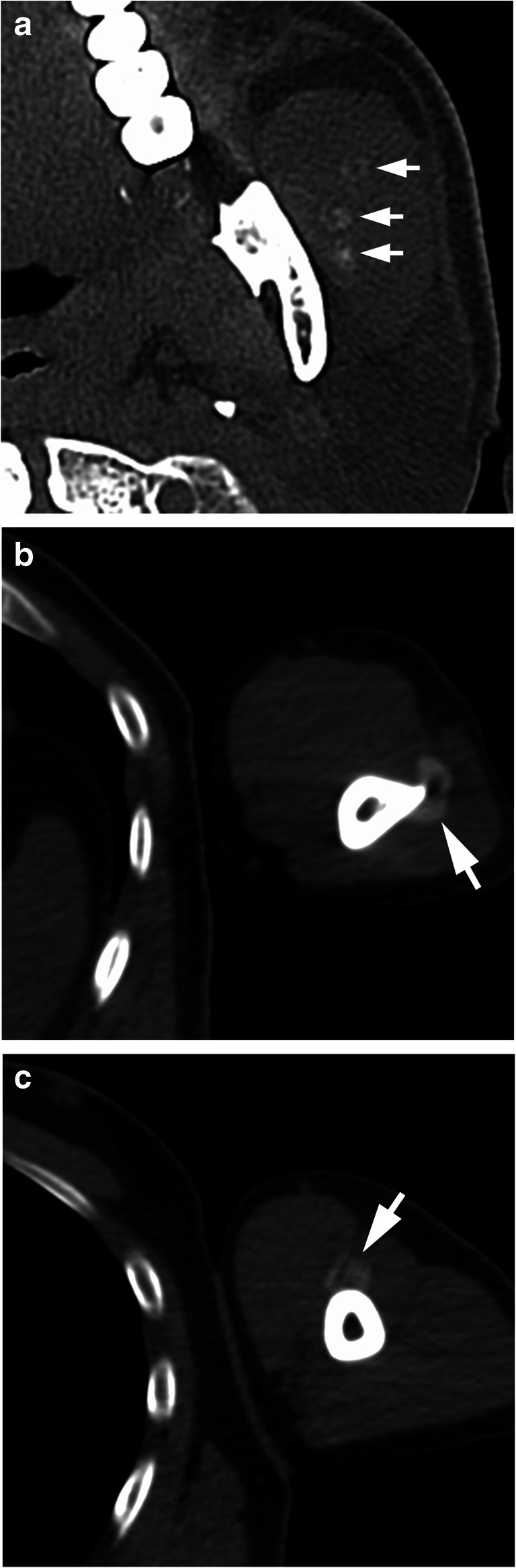
Conventional axial computed tomography images with bone-window setting show faint but circled calcifications in the **a** left masseter (arrows), **b** triceps brachii (arrow), and **c** biceps brachii (arrow) muscles

In a detailed medical interview after FDG-PET/CT, the patient reported a violent fall from a jet ski approximately 4 weeks previously that had injured his entire body, particularly the left side of his chest; however, no symptoms had manifested at the time. Subsequently performed contrast-enhanced whole-body CT revealed swelling of the left masseter muscle with no evidence of any internal tumor-like enhancement. Notably, faint but circled calcified foci at the left masseter were visible on bone-window CT (Fig. 3a). CT also revealed circled calcification at the two sites of brachial muscle; triceps brachii (Fig. 3b) and biceps brachii (Fig. 3c), and multiple fractures of the left zygomatic arch, left 8th rib, and left transverse process of L3. Increased attenuation and irregular striation within the subcutaneous adipose tissue were observed in the limbs. Cutaneous biopsy of the left lower leg revealed superficial perivascular dermatitis with healed septal panniculitis. Subsequently, various blood tests were performed, but no apparent signs of any specific inflammatory disease, systemic disease, or malignancy were observed.

**Fig. 4:**
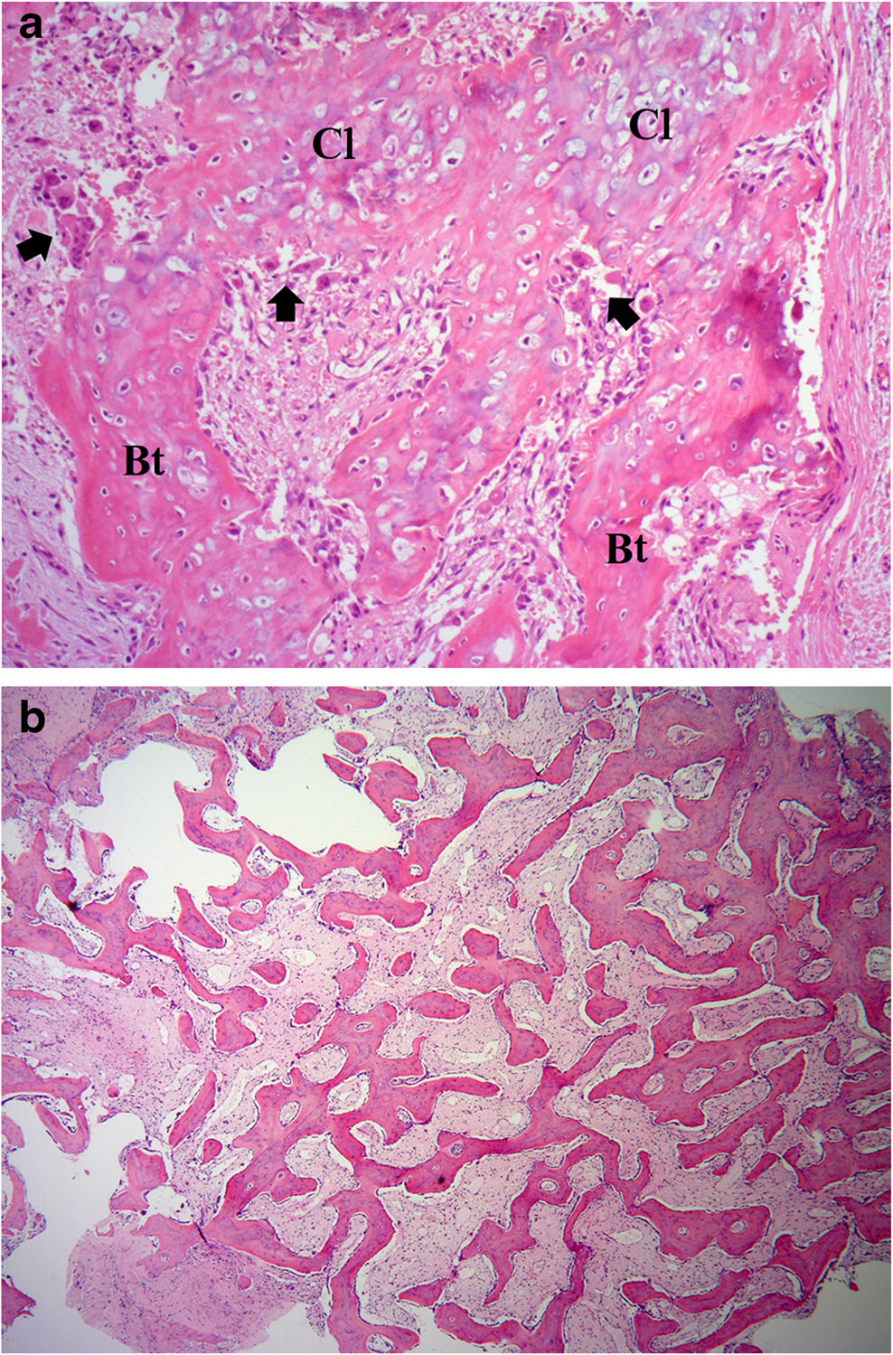
Histology of the excised intramuscular mass. **a** Immature bone trabeculae (Bt) in connection with cartilage (Cl) are rimmed by osteoblasts. Osteoclasts are arranged along the surface of transitional regions (arrows) (hematoxylin & eosin stain, × 100). **b** Massive ossification composed of immature irregular bone trabeculae is seen in the fibrous connective tissue (hematoxylin & eosin stain, × 40)

Based on these findings and the recent traumatic episode, TMOs of the masseter and brachial muscles with multiple fractures and subcutaneous inflammation throughout the limbs were strongly suspected. As the trismus remained unaltered, surgical excision of the mass in the left masseter muscle was performed. The intramuscular solid mass was histologically composed of fibrous connective tissue containing several foci of different ossifications. Peripheral trabecular ossification, including cartilaginous nodular cores, was predominant. The immature bone trabeculae that transition out of the cartilage were rimmed by plump osteoblasts, and osteoclasts were arranged along the surface of the transitional regions. This ossification pattern was reminiscent of endochondral bone formation (Fig. 4a). Massive immature bone spicules accompanied with disappearance of cartilage were also found in the fibrous connective tissue (Fig. 4b). The osteoblasts and chondrocytes were not atypical. Small foci of granulomatous lesions containing degenerated myocytes, macrophages, and lymphocytes were observed. These histopathological findings were consistent with MO and confirmed the diagnosis of TMO. Trismus improved after surgery, and re-occurrence was not detected over a follow-up period of 2 years.

## Discussion

The pathogenesis of MO has not been fully elucidated, but it is considered that heterotopic ossification is induced by pluripotent mesenchymal stem cells; these cells are derived from vascular endothelial cells via endothelial-mesenchymal transition and are capable of producing cartilage and bone [3]. The process can be divided into three stages: early, intermediate, and mature. The early stage, which occurs in the first 4 weeks after the injury, is the inflammatory phase. Early MO without ossification or calcification can easily be misdiagnosed as malignancy or infection. The intermediate stage, occurring 4–8 weeks after the injury, involves bone formation and is visible on conventional X-ray imaging or CT. Finally, the mature stage, which lasts for several months, is the formation of a mature peripheral zone with lamellar bone. Subsequently, bone regression occurs and surgical excision is undertaken depending on the patient’s symptoms.

Herein, we report a case of multiple TMOs in the masseter, triceps brachii, and biceps brachii muscles detected by FDG-PET/CT. At the time of the first consultation, MRI and FDG-PET/CT were initially performed because the patient had been suspected to have a malignant tumor by a previous doctor, due to the presence of a progressive and painless hard mass in the left masseter region. Moreover, the traumatic episode with whole-body contusion that occurred 4 weeks prior had not been disclosed as the patient had noticed no symptoms other than trismus. Panoramic tomography did not detect calcifications in the masseter muscle. If the traumatic episode had been disclosed at the first consultation, other conventional radiographs such as posterior-anterior projection or whole-body CT, which detect multiple TMOs more efficiently, would have been undertaken earlier.

MRI was the first imaging modality undertaken to evaluate the left cheek mass in the present case. The MRI features of MO vary depending on the maturity of the lesion [2, 5, 6]. In the early stage, before calcification becomes evident on plain X-ray images, T1-weighted images show isointensity to the muscle, and only a mass effect appears. On one hand, T2-weighted images demonstrate inflammatory edematous changes with various high signal intensities. In the present case, which was in the intermediate stage, faint calcifications were difficult to identify on MRI. On the other hand, a tumor-like signal intensity was found on the fat-saturated T2-weighted image (Fig. 1b) and dynamic contrast-enhanced MRI (Fig. 1c). Time-intensity curves after dynamic contrast-enhancement studies were suggestive of inflammation, but as these were not diagnostic, malignancy could not be ruled out.

In our case, FDG-PET/CT detected multiple TMOs in distinct locations. Few studies have reported FDG uptake in MO lesions [7–9]. FDG accumulates at the site of MO due to inflammation, mimicking malignant neoplasms. In general, FDG-PET/CT is performed when the affected lesion is suspected or diagnosed as malignant, in order to explore any other abnormalities. However, this imaging technique often fails to diagnose benignity, malignancy, or inflammation, hindering a definitive diagnosis. Multiple FDG uptakes around the whole body usually suggest the presence of multiple metastases, lymphoma, collagen disease, or sarcoidosis. In our case, the muscles, bones, and subcutaneous adipose tissues throughout the body showed multiple high-uptake regions on FDG-PET/CT, with the same uptake levels typically seen in malignancy. After subsequent examinations, including histopathology, the FDG uptakes were considered to have been caused by inflammation as a result of the Jet Ski trauma. High uptakes were also observed in the bilateral axillary lymph nodes, which enlarged from reactive changes due to the inflammation of the upper arms. It should be noted that whole-body contusion causes multiple FDG uptakes across the whole body, as reported here. Among them, circular calcification corresponding to the muscular FDG uptake is suggestive of MO; however, it is not diagnostic if the traumatic episode is unclear. The diagnostic imaging and surgical excision of the present case were performed during the intermediate stage according to temporal division. The histological appearance of MO also varies between stages. Our histological findings, predominantly cartilage, and immature bone formation, also indicated an intermediate stage [3]. These histopathological findings were consistent with MO and confirmed the diagnosis of TMO.

Only a few reports have described multiple occurrences of MO [10–12]. In such cases, multiple MOs exist bilaterally in the skeletal muscles, such as the deltoid [10] or muscles around the neck of the femur [11]. Another case of multiple MO involved the adjacent masticatory muscles [12]. In our case, multiple TMOs were not found bilaterally but far apart in the masseter and brachial muscles. Though the calcifications in the brachial muscles observed on CT were not histologically proven, both the masseter and brachial muscle lesions were strongly suspected to be TMOs according to the clinical and imaging findings. To the best of our knowledge, cases of multiple TMOs found in different muscles that are located far apart are rare, and this may be the first report.

The differential diagnosis of MO or TMO includes many extraosseous tumoral and non-tumoral diseases [2, 3], and to avoid unnecessary surgical procedures, it is crucial to differentiate these lesions from malignant soft-tissue tumors. However, it is sometimes difficult to verify the diagnosis of TMO, and especially, to differentiate it from malignant lesions through clinical examination and diagnostic imaging as shown in our case. Thus, if malignancy is not ruled out, additional histopathological examination is necessary to confirm the diagnosis.
